# Human metabolism and pharmacological profiling of protonitazepyne and metonitazepyne, two highly potent nitazenes: prediction of main metabolite activity based on µ-opioid receptor docking simulations

**DOI:** 10.1007/s00204-025-04163-4

**Published:** 2025-10-31

**Authors:** Diletta Berardinelli, Omayema Taoussi, Duygu Yeşim Ovat, Simona Pichini, Benedikt Pulver, Volker Auwärter, Francesco Paolo Busardò, Giuseppe Basile, Emiliano Laudadio, Jeremy Carlier

**Affiliations:** 1 Department of Biomedical Sciences and Public Health, Marche Polytechnic University, Via Tronto 10/a, 60126 Ancona, AN Italy; 2 Toxicology and Pharmaceutical Science, Institute On Drug Abuse, Ege University, Izmir, Türkiye; 3 National Centre On Addiction and Doping, National Institute of Health, Rome, Italy; 4 Forensic Toxicology, Institute for Legal Medicine, Medical Center, Faculty of Medicine, University of Freiburg, University of Freiburg, Freiburg, Germany; 5 Department of Science and Engineering of Matter, Environment and Urban Planning, Marche Polytechnic University, Ancona, Italy

**Keywords:** Toxicology, Pharmacokinetics, Pharmacodynamics, Metabolism, Molecular dynamics, Opioids, Novel psychoactive substances

## Abstract

Nitazenes have recently surfaced the illicit opioid market, causing numerous intoxications and fatalities. *N*-Pyrrolidino derivatives, protonitazepyne and metonitazepyne, have circulated since 2023 and have been involved in overdose intoxications. Their pharmacological properties remain largely unknown. However, pharmacokinetic/dynamic data are crucial for clinicians and toxicologists to manage intoxications and interpret legal cases. Protonitazepyne and metonitazepyne metabolism was assessed using human hepatocyte incubations and blood/urine from an intoxication case; samples were analyzed with liquid chromatography–high-resolution mass spectrometry and software-aided data mining. µ-(MOR), κ-(KOR), and δ-(DOR) opioid receptor activation was assessed using a GTP G_i_ binding assay. MOR docking was simulated with UCSF Chimera and AutoDockSuite. Pharmacological relevance of major metabolites was predicted through in silico MOR docking. Major metabolites were produced through nitroreduction, pyrrolidine *N*-dealkylation, and oxidation to *N*-butanoic acid and *O*-dealkylation. Protonitazepyne and metonitazepyne potencies at MOR were 3.7 and 11.5 nmol L^−1^, respectively; efficacies were 154 and 101%. Partial agonism and low potency were observed at KOR/DOR. In silico inhibition constants at MOR for protonitazepyne, 5-amino-protonitazepyne, metonitazepyne, and 5-amino-metonitazepyne were 0.68, 11.45, 1.98, and 2,050 nmol L^−1^, respectively. Protonitazepyne and metonitazepyne are MOR-selective full agonists, with potencies about seven and two times higher than fentanyl. These nitazenes present significant health risks through central nervous system/respiratory depression. Their primary metabolites showed lower/marginal in silico MOR affinity, suggesting they might be pharmacologically active, albeit to a much lesser extent than the parent compounds. We propose 5-amino derivatives (blood) and *N*-butanoic acid derivatives (urine) as biomarkers for detecting consumption.

**Supplementary Information:**

The online version contains supplementary material available at 10.1007/s00204-025-04163-4.

## Introduction

Nitazenes (2-benzylbenzimidazole analogs) make up a class of new psychoactive substances (NPSs) inducing opioid-like euphoria, analgesia, and anesthetic effects. These compounds have been misused as substitutes for opiates or other opioids since 2019, owing to their lower cost and to circumvent drug laws and analytical detection (EMCDDA 2024). Nitazenes are typically more potent than heroin and even fentanyl, and can cause massive central nervous system (CNS) and respiratory depression; they have already been involved in hundreds of overdose fatalities worldwide, either alone or in combination with other drugs, notably benzodiazepines (“benzo-dope” mixture) and tranquilizers (“tranq-dope”) (Montanari et al. 2022; Smith et al. 2023; Di Trana et al. 2023; Berardinelli et al. 2024; Taoussi et al. 2024; Floresta et al. 2025). Nitazenes present challenges to clinicians and toxicologists: (1) they are typically active at low blood concentrations (~ ng mL^−1^) and are rapidly metabolized, making them undetectable in biological matrices after a short period of time; (2) some metabolites are active, contributing to the psychoactive effects and potentially prolonging their duration (Ujváry et al. 2021; Vandeputte et al. 2021; Monti et al. 2024); (3) metabolic degradation may occur through highly polymorphic enzymes, which can influence metabolite production and potentially alter their overall effects in an individual (Jadhav and Fasinu 2024). Understanding the pharmacokinetics and pharmacodynamics of nitazenes, through toxicology research, is, therefore, crucial for accurately identifying positive cases and interpreting parent compound and metabolite concentrations in biological samples within clinical and forensic settings. This, in turn, is essential for managing intoxications, treating patients and interpreting legal cases. However, speed is critical to keep pace with the illicit market.

Protonitazepyne and metonitazepyne are *N*-pyrrolidino-substituted nitazenes that were first identified on the US drug market in 2023, and in the European Union a few months later; seizures were reported in Slovenia, Latvia, Estonia, Denmark, Germany, Austria, Italy, Greece, and Ireland (Center for Forensic Science Research and Education (CFSRE) 2021; European Database on New Drugs 2023). Both compounds have been controlled as Schedule I substances in the USA since October 2024 (Drug Enforcement Administration 2024). They are not explicitly banned in most other countries, although they may fall under local analog or specific NPS legislation. The two nitazenes have been detected in several toxicology cases in the US (Center for Forensic Science Research and Education (CFSRE) 2023a, Center for Forensic Science Research and Education (CFSRE) 2023b). However, no data are currently available regarding their concentrations in biological samples. In vitro studies of µ-opioid receptor (MOR) activation have shown that both protonitazepyne and metonitazepyne are substantially more potent than fentanyl (Kozell et al. 2024; De Vrieze et al. 2024), and concentrations in the ng/mL range are expected in blood following recreational use or intoxication (United Nations 1961). There is currently no data available on the two drugs’ pharmacokinetics.

The present study aimed to assess the metabolism of protonitazepyne and metonitazepyne to identify metabolite biomarkers of consumption applicable in clinical and forensic settings. In addition, potentially pharmacologically active metabolites were investigated. For this purpose, human hepatocyte incubations, as well as protonitazepyne-positive blood and urine samples, were analyzed using liquid chromatography–high-resolution tandem mass spectrometry (LC–HRMS/MS) combined with software-aided data mining. Protonitazepyne and metonitazepyne activity at MOR, δ- (DOR), and κ- (KOR) opioid receptors were evaluated using a GTP Gi binding assay to investigate their pharmacological effects. Finally, molecular docking of protonitazepyne, metonitazepyne, and their main metabolite in blood was conducted at MOR to predict their binding affinity and anticipate potential activity/toxicity without relying on costly, time-consuming, and analytical standard-dependent laboratory experiments.

## Materials and methods

### Chemicals and reagents

Protonitazepyne, metonitazepyne, fentanyl, SNC-80, and U-50488 pure standards were bought from Cayman Chemical (Ann Arbor, Michigan, USA). LC–MS-grade acetonitrile, water, and formic acid (FA) were obtained from Carlo Erba (Cornaredo, Italy). Williams’ medium E, HEPES buffer (2-[4-(2-hydroxyethyl)−1- piperazinyl]ethanesulfonic acid), *l*-glutamine, ammonium acetate, and β-glucuronidase from limpets (*P. vulgata*) were obtained from Sigma Aldrich (Milan, Italy). Supplemented Williams’ medium E (SWM) was prepared by dissolving HEPES and *l*-glutamine at 2 and 20 mmol/L, respectively, in Williams’ medium E. Pooled cryopreserved human hepatocytes (HEP) from ten fully anonymized donors were purchased from Lonza (Basel, Switzerland); human tissue is acquired from tissue recovery agencies, tissue suppliers, and Lonza-managed donor programs that perform tissue recovery and donor informed consent in accordance with processes approved by an Institutional Review Board. MOR, DOR, and KOR membranes and GTP Gi binding assay kits were purchased from Revvity (Milan, Italy).

### Protonitazepyne and metonitazepyne metabolism

#### Hepatocyte incubation

Protonitazepyne and metonitazepyne were individually incubated with HEP following our in-house protocol (Taoussi et al. 2024). Briefly, 250 µL of 20 µmol L^−1^ protonitazepyne and metonitazepyne in SWM were incubated at 37 °C for 3 h with 2 × 10^6^ viable cells/mL in SWM in 24-well culture plates. The reactions were stopped with 500 µL of ice-cold acetonitrile and centrifugation for 10 min, 15,000 g. Samples were stored at –80 °C until analysis. Negative and positive controls were incubated under the same conditions for 0 and 3 h to rule out interference and non-specific reactions and confirm metabolic activity.

#### Authentic biological samples

Femoral blood and urine from a fatal intoxication involving protonitazepyne were collected at the autopsy and analyzed to confirm the metabolites identified in vitro. The data were obtained as part of routine forensic investigations and fully anonymized. Therefore, in accordance with German and Italian legislation, informed consent and ethics committee approval were not required.

#### Sample preparation

After thawing at room temperature, 100 µL HEP incubate was mixed with 100 µL acetonitrile and centrifuged for 10 min, 15,000 g, at room temperature. The supernatants were evaporated to dryness under nitrogen at 37 °C. The dried residues were reconstituted with 100 µL of 0.1% FA in water:0.1% FA in acetonitrile 90:10 (v/v), then centrifuged again under the same conditions. The supernatants were transferred into vials with glass inserts prior to analysis with LC–HRMS/MS.

One hundred µL blood or urine were mixed with 200 µL acetonitrile and centrifuged for 10 min, 15,000 g, at room temperature. The supernatants were evaporated to dryness under nitrogen at 37 °C. The dried residues were reconstituted with 100 µL of 0.1% FA in water:0.1% FA in acetonitrile 95:5 (v/v), then centrifuged again under the same conditions. The supernatants were transferred into vials with glass inserts prior to analysis with LC–HRMS/MS.

To investigate glucuronide conjugations, 100 µL urine was mixed with 10 µL of 10 mol/L ammonium acetate at pH 5.0, and 100 µL β-glucuronidase (5,000 units), and incubated for 90 min at 37 °C; a negative control with 100 µL water instead of β-glucuronidase was also prepared. Four hundred µL ice-cold acetonitrile was added to the mixtures for protein precipitation. After centrifugation for 10 min, 15,000 g, at room temperature, the supernatants were evaporated to dryness under nitrogen at 37 °C and reconstituted in 100 µL of 0.1% FA in water:0.1% FA in acetonitrile 95:5 (v/v). After centrifugation under the same conditions, the supernatants were transferred into vials with glass inserts prior to analysis with LC–HRMS/MS.

#### LC–HRMS/MS analysis

The analyses were performed with a DIONEX UltiMate 3000 chromatographic system coupled to a Thermo Scientific Q-Exactive quadrupole-Orbitrap mass spectrometer equipped with a heated electrospray ionization (HESI) source. LC–HRMS/MS conditions were the same as those previously described for metabolite identification of isotonitazene and structural analogs to identify shared metabolites, with minor modifications (Berardinelli et al. 2024; Taoussi et al. 2024) 1) The ramped normalized collision energy was optimized for the analysis of protonitazepyne and metonitazepyne to generate relevant fragments for structure elucidation (40, 55, and 80%); 2) Inclusion lists of putative metabolites were used to prioritize HRMS/MS fragmentation based on in silico predictions and postulations (Krotulski et al. 2021a, b; Di Trana et al. 2023; Murari et al. 2024; Berardinelli et al. 2024; Taoussi et al. 2024; Ameline et al. 2024; Jadhav and Fasinu 2024; Monti et al. 2024) (Supplemental Tables S1 and S2).

#### Software-aided metabolite identification

LC–HRMS/MS data were screened with Thermo Scientific Compound Discoverer, as previously detailed (Di Trana et al. 2021). Settings were the same as those described for the metabolite identification of isotonitazene and structural analogs (Berardinelli et al. 2024; Taoussi et al. 2024) with a specific list of theoretical metabolites based on in silico predictions and postulations (Krotulski et al. 2021a, b; Di Trana et al. 2023; Murari et al. 2024; Berardinelli et al. 2024; Taoussi et al. 2024; Ameline et al. 2024; Jadhav and Fasinu 2024; Monti et al. 2024), and generated according to the settings displayed in Supplemental Table S3.

### In vitro*** opioid receptor activation (GTP G***_***i***_*** binding assay)***

MOR, KOR, and DOR activation by protonitazepyne and metonitazepyne was assessed using an HTRF®-based GTP G_i_ binding assay, designed to evaluate the activation of G_i_ protein-coupled receptors with high sensitivity and specificity (low background), while avoiding the need for radioligands entailing specific regulatory and safety requirements (Principle 2023). The assay was performed following our in-house protocol (Berardinelli et al. 2025). Protonitazepyne, metonitazepyne, and controls (MOR, fentanyl; KOR, U-50488; DOR, SNC-80) were incubated overnight at room temperature with a supplemented stimulation buffer with optimized GDP and magnesium chloride concentrations, a detection reagent mix of equal volumes of europium cryptate and d2-labeled antibody, and human MOR, KOR, or DOR membrane preparation (total volume, 20 µL). Protonitazepyne, metonitazepyne, and controls’ concentrations ranged from 10–5 to10-11 mol L^−1^; each concentration was tested in duplicates, and the experiments were conducted in triplicates. Non-specific binding was evaluated using a non-hydrolyzable GTPγS at saturation to measure the assay background signal. The fluorescence resonance energy transfer (FRET) signal was detected using a Multilabel Plate Reader (PerkinElmer), and the fluorescence ratio at 665 and 620 nm was calculated (delay, 100 μs; total window time, 200 μs). All values were normalized to the maximum signal of the reference compounds for each receptor. Concentration–response curves were generated using GraphPad Prism (v. 10.2.3) with a three-parameter fit to determine the potency (EC_50_) and efficacy (E_max_) of the compounds.

### In silico MOR docking

Considering in vitro opioid receptor activation preliminary results, receptor docking was only assessed at MOR.

The three-dimensional structure of protonitazepyne, metonitazepyne, their main metabolites in blood, and controls (morphine and fentanyl) were generated and minimized using UCSF Chimera. MOR crystallographic structure was obtained by the 5c1m pdb file (Pettersen et al. 2004; Munro 2023). Ligand–MOR interactions were investigated using AutoDock Suite 4.2 (Morris et al. 2009), with AutoDockTools to add polar hydrogen atoms and partial charges to the receptor and ligands, Addsol to assign MOR atomic solvation parameters and fragmental volumes, Autotors to assign ligands’ flexible torsions (all dihedral angles were allowed to rotate freely), and Autogrid to generate affinity grid fields.

A grid field of 50 × 58 × 44 Å and the resulting docked conformations were clustered into families of similar binding modes, with a root mean square deviation (RMSD) clustering tolerance of 2 Å. The lowest and the most populated docking conformations were considered as the most stable orientations. The binding energy, representing the sum of the intermolecular contributions and the internal energy of the ligand (Den Otter and Briels 1998), was calculated by an empirical free-energy force field with a Lamarckian genetic algorithm (LGA), and can be translated into a simulated inhibition constant (K_i_) through the thermodynamic law ∆G =  − RT × ln(K_i_).

The binding poses with the highest binding affinity and population percentage were analyzed using molecular dynamic (MD) simulations to assess binding stability over time and the ligand and receptor functional groups involved in binding (Den Otter and Briels 1998). A membrane composed of 142 POPC (1-palmitoyl-2-oleoyl-sn-glycero-3-phosphocholine) lipids was modeled to stabilize the MOR active conformation in its native environment; MOR was inserted inside the membrane using the correct coordinates obtained by positioning of proteins in membranes (PPM) server (Lomize et al. 2012). A simulation box of 7.786 × 7.786 × 8.278 nm was generated using CHARMM-GUI, and periodic boundary conditions were used along all axes (Jo et al. 2008). To reach physiological conditions at 0.15 mol L^−1^ NaCl, the simulation box was solvated by 7,100 TIP3 water molecules, 15 Na^+^ ions, and 30 Cl^–^ counterions (Mark and Nilsson 2001). Each ligand–MOR complex underwent a minimization step followed by six equilibration cycles. A total of 200 ns of MD simulations under semi-isotropic conditions were performed for the production phase, maintaining constant number of molecules, pressure, and temperature. All the simulations were performed using GROMACS 2023.3 and CHARMM36 force field. Finally, the difference between initial and final positions of a ligand within the binding site was analyzed by computing the RMSD over time, using Visual Molecular Dynamics (VMD) and UCSF Chimera software (Humphrey et al. 1996; Pettersen et al. 2004; Huang and Mackerell 2013).

## Results

### In vitro and in vivo metabolism of protonitazepyne and metonitazepyne

#### LC–HRMS/MS fragmentation patterns

**Fig. 1:**
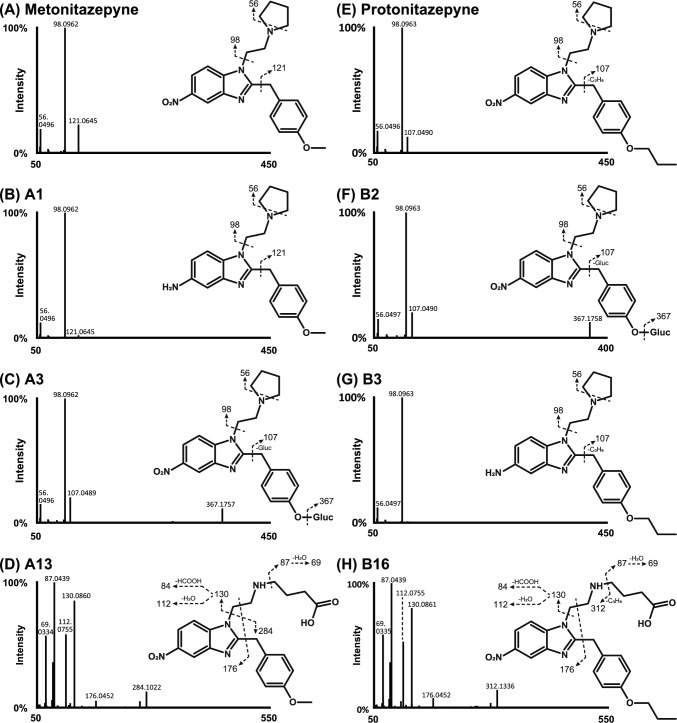
HRMS/MS spectra of metonitazepyne (**A**), and major metabolites A1 [5-amino-metonitazepyne (**B**)], A3 [*O*-desmethyl metonitazepyne glucuronide (**C**)], and A13 [*N*-butanoic acid metonitazene (**D**)], as well as their suggested fragmentation patterns. HRMS/MS spectra of protonitazepyne (**E**), and major metabolites B2 [*O*-desmethyl-protonitazepyne glucuronide (**F**)], B3 [5-amino-protonitazepyne (**G**)], and B16 [*N*-butanoic acid-protonitazene (**H**)], as well as their suggested fragmentation patterns. A3 = B2; Gluc, glucuronide

Protonitazepyne ([M + H]^+^ at *m/z* 409.2224, eluting at 17.26 min) and metonitazepyne ([M + H]^+^ at *m/z* 381.1912, eluting at 14.28 min) were only detected in positive-ionization mode, and displayed a similar HRMS/MS spectrum with few fragments (Fig. 1), consistent with the fragmentation of structural analogs under the same analytical conditions (Berardinelli et al. 2024; Taoussi et al. 2024). For both compounds, the predominant fragment corresponded to the *N*-ethyl pyrrolidine side chain at *m/z* 98.0964 ± 5 ppm (C_6_H_12_N^+^), which further yielded a minor fragment at *m/z* 56.0495 ± 5 ppm corresponding to an *N*-propyl group (C_3_H_6_N^+^). Fragment at *m/z* 121.0645 in metonitazepyne corresponded to the 1’-methyl-4’-methoxybenzyl side chain (C_8_H_9_O^+^), which was further fragmented to *m/z* 107.0490 in protonitazepyne due to propyl loss (C_7_H_7_O^+^).

#### In vitro and in vivo findings

Metonitazepyne and protonitazepyne LC–HRMS peak area was 70% and 80% reduced, respectively, after 3 h incubation with HEP. Seventeen metabolites were identified in 3-h incubates with metonitazepyne (A1-A17, ordered by ascending retention time) and protonitazepyne (B1-B17). The metabolic transformations were similar for both compounds, with major reactions including pyrrolidine hydroxylation and oxidation (presumably leading to γ-lactam formation (Carlier et al. 2021)), *N*-dealkylation of the pyrrolidine ring to form the *N*-butanoic acid derivative, and *O*-dealkylation followed by *O*-glucuronidation. Other reactions included hydroxylation, oxidative deamination, nitroreduction, *N*-glucuronidation, and sulfation. Complete in vitro results are compiled in Table 1.

**Table 1:** Metabolic transformation, elemental composition, retention time (RT), experimental accurate mass of molecular ion, deviation from theoretical accurate mass, and liquid chromatography–high-resolution mass spectrometry peak area of metonitazepyne and metabolites (A1–17), as well as protonitazepyne and metabolites (B1–17) in positive- and negative-ionization mode after 3 h incubation with human hepatocytes and blood and urine from an intoxication case

ID	Biotransformation	Elemental composition	RT, min	*m/z* [M + H]^+^ [M-H]^−^	Mass error, Δppm	Hepatocyte peak area at T_3h_: [M + H]^+^ [M-H]^−^	Blood peak area: [M + H]^+^ [M-H]^−^	Urine peak area: [M + H]^+^ [M-H]^−^	
								Without hydrolysis	With hydrolysis
A1	Nitro reduction	C_21_H_26_N_4_O	4.84	351.2180 ND^a^	0.18 ND	3.5 × 10^6^ ND	NA^b^	NA	NA
A2	O-Dealkylation + Nitro reduction + Oxidation (pyrrolidine)	C_20_H_23_N_4_O_2_	6.89	351.1816 ND	0.13 ND	1.2 × 10^6^ ND	NA	NA	NA
A3	*O*-Dealkylation + *O*-Glucuronidation	C_26_H_31_N_4_O_9_	8.37	543.2083 541.1957	−0.65 3.14	2.1 × 10^7^ 9.4 × 10^6^	NA	NA	NA
A4	Hydroxylation + *O*-Glucuronidation	C_27_H_32_N_4_O_10_	8.81	573.2195 ND	0.66 ND	5.7 × 10^5^ ND	NA	NA	NA
A5	*O*-Dealkylation + Hydroxylation (pyrrolidine)	C_20_H_23_N_4_O_4_	9.57	383.1714 ND	0.05 ND	3.1 × 10^6^ ND	NA	NA	NA
A6	*N*-Dealkylation to *N*-butanoic acid + *O*-Dealkylation	C_20_H_23_N_4_O_5_	9.82	399.1660 397.1533	−0.74 3.67	1.1 × 10^7^ 4.3 × 10^6^	NA	NA	NA
A7	Nitro reduction + Oxidation (pyrrolidine)	C_21_H_25_N_4_O_2_	10.44	365.1974 ND	0.54 ND	1.1 × 10^7^ ND	NA	NA	NA
A8	*O*-Dealkylation	C_20_H_23_N_4_O_3_	10.49	367.1765 365.1631	0.09 2.97	1.9 × 10^7^ 3.6 × 10^6^	NA	NA	NA
A9	*O*-Dealkylation + Oxidative deamination	C_16_H_15_N_3_O_4_	12.01	314.1128 ND	−2.33 ND	7.4 × 10^6^ ND	NA	NA	NA
A10	Hydroxylation (pyrrolidine) + *O*-Glucuronidation	C_27_H_32_N_4_O_10_	12.14	573.2181 ND	−1.78 ND	1.8 × 10^6^ ND	NA	NA	NA
A11	*N*,*N*-Didealkylation	C_17_H_18_N_4_O_3_	12.25	327.1447 ND	−1.43 ND	4.3 × 10^6^ ND	NA	NA	NA
A12	Hydroxylation (pyrrolidine)	C_21_H_25_N_4_O_4_	13.12	397.1865 ND	−1.34 ND	6.3 × 10^7^ ND	NA	NA	NA
A13	*N*-Dealkylation to *N*-butanoic acid	C_21_H_25_N_4_O_5_	13.46	413.1813 411.1683	−1.56 2.21	8.5 × 10^7^ 3.0 × 10^7^	NA	NA	NA
**Parent**	**Metonitazepyne**	**C**_**21**_**H**_**25**_**N**_**4**_**O**_**3**_	**14.28**	**381.1912** **ND**	**−2.41** **ND**	**2.2 × 10**^**8**^ **ND**	NA	NA	NA
A14	*N*-Dealkylation to *N*-butanal	C_21_H_25_N_4_O_4_	14.39	399.2024 ND	0.35 ND	6.2 × 10^5^	NA	NA	NA
A15	Hydroxylation	C_21_H_25_N_4_O_4_	14.85	397.1872 ND	0.42 ND	9.4 × 10^6^ ND	NA	NA	NA
A16	*O*-Dealkylation + Oxidation (pyrrolidine)	C_20_H_21_N_4_O_4_	15.33	381.1561 ND	0.97 ND	1.2 × 10^7^ ND	NA	NA	NA
A17	Oxidation (pyrrolidine)	C_21_H_23_N_4_O_4_	17.99	395.1706 ND	−1.98 ND	7.0 × 10^7^ ND	NA	NA	NA
B1	*O*-Dealkylation + Sulfation	C_20_H_22_N_4_O_6_S	3.27	447.1340 ND	1.61 ND	4.9 × 10^6^ ND	ND ND	ND ND	ND ND
B2	*O*-Dealkylation + *O*-Glucuronidation	C_26_H_31_N_4_O_9_	8.38	543.2084 541.1943	−0.28 0.55	6.4 × 10^7^ 3.1 × 10^7^	ND ND	2.6 × 10^7^ 9.4 × 10^6^	ND ND
B3	Nitro reduction	C_23_H_30_N_4_O	8.70	379.2492 ND	−0.10 ND	2.3 × 10^6^ ND	6.7 × 10^6^ ND	3.5 × 10^6^ ND	4.5 × 10^6^ ND
B4	*O*-Dealkylation + Oxidative deamination + *O*-Glucuronidation	C_22_H_23_N_3_O_10_	9.29	490.1459 ND	0.57 ND	3.6 × 10^6^ ND	ND ND	ND ND	ND ND
B5	*N*-Dealkylation to *N*-butanoic acid + *O*-Dealkylation	C_20_H_23_N_4_O_5_	9.85	399.1662 397.1532	−0.24 3.67	9.6 × 10^6^ 3.8 × 10^6^	ND ND	ND ND	ND ND
B6	*O*-Dealkylation	C_20_H_23_N_3_O_4_	10.52	367.1765 ND	0.09 ND	1.8 × 10^7^ ND	ND ND	1.6 × 10^6^ ND	5.2 × 10^7^ ND
B7	*O*-Dealkylation + Oxidative deamination	C_16_H_15_N_3_O_4_	12.04	314.1129 312.0994	−2.01 1.35	1.4 × 10^7^ 4.2 × 10^6^	ND ND	6.8 × 10^5^ ND	9.5 × 10^6^ ND
B8	*N*-Dealkylation to *N*-butanoic acid + Hydroxylation	C_23_H_29_N_4_O_6_	12.04	457.2075 ND	−1.45 ND	1.5 × 10^7^ ND	ND ND	3.9 × 10^6^ ND	4.2 × 10^6^ ND
B9	*O*-Dealkylation + Oxidation (pyrrolidine) + *O*-Glucuronidation	C_26_H_28_N_4_O_10_	12.32	557.1881 ND	0.50 ND	2.6 × 10^6^ ND	ND ND	ND ND	ND ND
B10	Hydroxylation	C_23_H_29_N_4_O_4_	12.56	425.2185 ND	0.39 ND	1.8 × 10^7^ ND	ND ND	3.5 × 10^6^ ND	3.5 × 10^6^ ND
B11	Nitro reduction + Oxidation (pyrrolidine)	C_23_H_29_N_4_O_2_	14.69	393.2287 ND	0.50 ND	2.4 × 10^7^ ND	3.3 × 10^6^ ND	2.5 × 10^6^ ND	2.9 × 10^6^ ND
B12	*O*-Dealkylation + Oxidation (pyrrolidine)	C_20_H_20_N_4_O_4_	15.35	381.1558 ND	0.18 ND	8.9 × 10^6^ ND	ND ND	ND ND	5.5 × 10^6^ ND
B13	*N*,*N*-Didealkylation	C_19_H_23_N_4_O_3_	16.29	355.1766 ND	0.37 ND	1.8 × 10^7^ ND	ND ND	ND ND	ND ND
B14	*N*-Glucuronidation	C_29_H_37_N_4_O_9_	16.46	585.2558 ND	0.50 ND	9.5 × 10^6^ ND	ND ND	ND ND	ND ND
B15	Hydroxylation (Pyrrolidine)	C_23_H_29_N_4_O_4_	16.62	425.2181 ND	−0.54 ND	2.6 × 10^7^ ND	ND ND	ND ND	ND ND
B16	*N*-Dealkylation to *N*-butanoic acid	C_23_H_29_N_4_O_5_	16.75	441.2123 439.1993	−2.14 1.38	3.2 × 10^8^ 1.4 × 10^8^	1.1 × 10^5^ 6.0 × 10^4^	1.1 × 10^7^ ND	2.6 × 10^7^ 5.7 × 10^6^
**Parent**	**Protonitazepyne**	**C**_**23**_**H**_**29**_**N**_**4**_**O**_**3**_	**17.26**	**409.2224** **ND**	**−2.49** **ND**	**3.4 × 10**^**8**^ **ND**	**1.2 × 10**^**6**^ **ND**	**1.4 × 10**^**7**^ **ND**	**8.7 × 10**^**6**^ **ND**
B17	Oxidation (pyrrolidine)	C_23_H_27_N_4_O_4_	19.78	423.2022 ND	−1.14 ND	9.8 × 10^7^ ND	5.8 × 10^5^ ND	8.7 × 10^5^ ND	7.8 × 10^5^ ND

^a^ND, not detected

^b^NA, not applicable

No additional metabolites were identified in protonitazepyne-positive urine and blood samples. Nine metabolites were found in non-hydrolyzed urine; pyrrolidine *N*-dealkylation to *N*-butanoic acid and *O*-despropylation followed by *O*-glucuronidation were major transformations, *N*-butanoic acid-protonitazene (B16) and *O*-despropyl-protonitazepyne glucuronide (B2) being predominant, similar to in vitro results. The only urinary glucuronide (B2) was completely cleaved after enzymatic hydrolysis, leading to a 30-fold increase in the signal of the corresponding unconjugated metabolite, *O*-despropyl-protonitazepyne (B6). Four metabolites were identified in blood; unlike HEP incubation results, nitroreduction was the predominant transformation, 5-amino-protonitazepyne (B3) being the main metabolite. Complete in vivo results are compiled in Table 1.

The extracted-ion chromatograms of metonitazepyne and protonitazepyne and metabolites after 3 h incubation with HEP and in the positive samples are displayed in Supplemental Fig. S1.

Structure elucidation of the main metabolites in protonitazepyne-positive samples is described below, with their HRMS/MS spectra being displayed in Fig. 1. Considering their similar in vitro metabolism, the corresponding metabolites for metonitazepyne are also described.

#### Major metabolite structure elucidation

The most intense metabolites in HEP for both metonitazepyne (A13, [M + H]^+^ at *m/z* 413.1813) and protonitazepyne (B16, [M + H]^+^ at *m/z* 441.2123) resulted from *N*-dealkylation followed by oxidation to a butanoic acid derivative (+ 2O, as indicated by the + 31.9900 Da ± 5 ppm mass shift from the parents); B16 was the second most intense metabolite in protonitazepyne-positive urine. Both A13 and B16 were also detected at a high intensity in negative-ionization mode due to the formation of a carboxylate anion ([M-H]^−^ at *m/z* 411.1683 and 439.1993, respectively). This transformation follows a classic metabolic pathway for pyrrolidines, involving oxidation at carbon 2 to yield a γ-lactam intermediate, which subsequently undergoes ring opening (Carlier et al. 2021). In positive-ionization mode, cleavage at the benzimidazole core generated a major fragment at *m/z* 130.0860 (C_6_H_12_NO_2_^+^) in both analogs, corresponding to the *N*-ethyl-*N*-butanoic side chain, and a minor one at *m/z* 284.1015 (C_15_H_14_N_3_O_3_^+^) and 312.1131 (C_17_H_18_N_3_O_3_^+^) for metonitazepyne and protonitazepyne, respectively; further neutral losses of water and formic acid from the *N*-ethyl-*N*-butanoic group produced fragments at *m/z* 112.0755 (C_6_H_10_NO^+^) and 84.0806 (C_5_H_10_N^+^), respectively. In addition, the butanoic acid group yielded a fragment at *m/z* 87.0439 (C_4_H_7_O_2_^+^), with subsequent water loss at *m/z* 69.0335 (C_4_H_5_O^+^).

*O*-Desalkylation of metonitazepyne (-CH_2_) and protonitazepyne (-C_3_H_6_) resulted in the same metabolite (A8 = B6, [M + H]^+^ at *m/z* 367.1765). A8 = B6 exhibited the same fragmentation pattern as protonitazepyne, with key fragments at *m/z* 56.0495 (*N*-propyl, C_3_H_6_N^+^), 98.0961 (*N*-ethylpyrrolidine, C_6_H_12_N^+^), and 107.0488 (1’-methyl-4’-hydroxybenzyl, C_7_H_7_O^+^), indicating that these functional groups remained unchanged. Further *O*-glucuronidation (+ C_6_H_8_O_6_) produced A3 = B2 at *m/z* 543.2083 ± 5 ppm. This was the third and second most intense metabolite in metonitazepyne and protonitazepyne incubations, respectively, while also being the predominant in protonitazepyne-positive urine. A3 = B2 displayed a fragmentation pattern similar to its non-conjugated precursor A8 = B6. The position of the glucuronide was confirmed by its susceptibility to β-glucuronidase cleavage, demonstrating that conjugation occurred at an oxygen rather than a nitrogen atom.

Metonitazepyne and protonitazepyne nitroreduction (-HO_2_) produced the 5-amino derivatives A1 ([M + H]^+^ at *m/z* 351.2180) and B3 ([M + H]^+^ at *m/z* 379.2492), respectively. These metabolites were minor in vitro, but B3 was preponderant in protonitazepyne-positive blood, in line with previous results from structural analogs incubated under the same conditions (Taoussi et al. 2024). A1 and B3 exhibited a fragmentation pattern similar to that of the corresponding parent compound, indicating that the side chains remained unchanged.

### In vitro opioid receptor activation of protonitazepyne and metonitazepyne

**Fig. 2:**
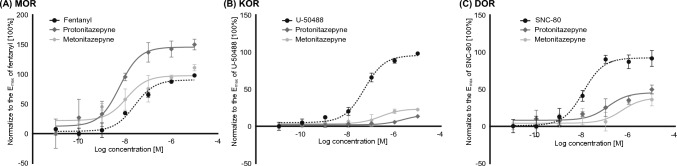
Metonitazepyne and protonitazepyne μ-(MOR) (**A**), κ-(KOR) (**B**), and δ-(DOR) (**C**) opioid activation profiles (normalized to fentanyl, U-50488, and SNC-80 signal). Data are presented as mean ± SEM, n = 3; FRET, fluorescence resonance energy transfer

The in vitro activation profiles of metonitazepyne and protonitazepyne at MOR, DOR, and KOR, represented by the normalized FRET signal intensity as a function of drug concentration, are shown in Fig. 2. Table 2 displays protonitazepyne and metonitazepyne in vitro EC_50_ and E_max_ at MOR, DOR, and KOR. Metonitazepyne and protonitazepyne potencies at MOR were approximately two and seven times higher than that of fentanyl, while displaying low potency at KOR and DOR.

**Table 2:** In vitro MOR, KOR, and DOR activation using a HTRF®GTP Gi binding assay, represented by their potency (EC_50_) and efficacy (E_max_) values (relative to fentanyl [MOR], U-50488 [KOR], SNC-80 [DOR]), and in silico binding energy, represented by their simulated inhibition constant (K_i_). 95% confidence intervals are given between parentheses

Compound	MOR				KOR		DOR	
	EC_50_, nmol L^−1^	E_max_, %	Binding energy, kcal/mol	Simulated K_i_, nM	EC_50_, nmol L^−1^	E_max_, %	EC_50_, nmol L^−1^	E_max_, %
Fentanyl	25.6 (10.1- 70.2)	100 (98–119)	−11.7	3.62	NA^a^	NA	NA	NA
Morphine	NA	NA	−11.2	3.85	NA	NA	NA	NA
U-50488	NA	NA	NA	NA	49.4 (29.6–80.1)	100 (98–113)	NA	NA
SNC-80	NA	NA	NA	NA	NA	NA	4.5 (2.1–9.7)	100 (89–108)
Protonitazepyne	3.7 (2.2–8.7)	154 (152–186)	−13.2	0.68	2530 (2311.2–2781.5)	14 (10–24)	425 (202.3–790.5)	56 (54–72)
5-Aminoprotonitazepyne	NA	NA	−10.3	11.4	NA	NA	NA	NA
Metonitazepyne	11.5 (6.8–14.6)	101 (84–120)	−12.3	1.98	257 (196.9–311.6)	22 (16–33)	266 (96.8–742.7)	36 (30–46)
5-Aminometonitazepyne	NA	NA	−7.76	2.05 × 10^3^	NA	NA	NA	NA

^a^NA, not applicable

### In silico MOR docking of protonitazepyne, metonitazepyne, and their main metabolites

#### Model validation

The crystal structure 5c1m shows MOR in its active conformation bound the high-affinity agonist BU72. A focused docking approach was employed to reproduce the binding mode observed in the crystallographic structure. The resulting pose closely matched the experimental binding mode, thereby validating our docking strategy (Supplemental Fig. S2).

#### MOR docking

Docking simulations were performed for metonitazepyne, protonitazepyne, 5-amino-metonitazepyne (A1), and 5-amino-protonitazepyne (B3). A mapping of the binding site is shown in Supplemental Fig. S2. This site consists of 19 amino acids spanning 5 of the 7 transmembrane helices (TMs) and appears oval, with TM1 and TM5 at the extremities; TM2 and TM4 were not involved in the binding. The site is heterogeneous, with polar, apolar, and charged amino acids.

**Fig. 3:**
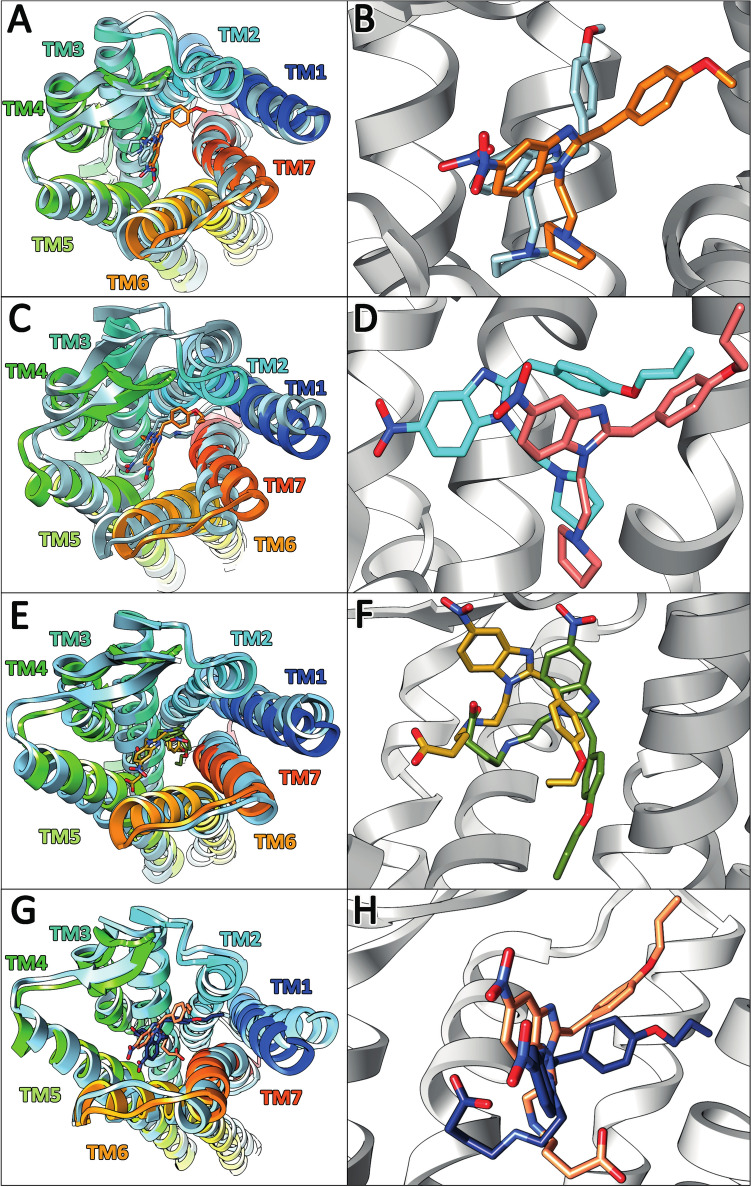
Analysis of MOR–ligand complexes: metonitazepyne-MOR complex (**A**) and focus on the binding site (**B**) (initial and final poses in orange and light blue sticks, respectively); 5-amino-metonitazepyne-MOR complex (**C**) and focus on the binding site (**D**) (initial and final poses in pink and light blue sticks, respectively); protonitazepyne-MOR complex (**E**) and focus on the binding site (**F**) (initial and final poses in goldenrod and green sticks, respectively); and protonitazepyne-MOR complex (**G**) and focus on the binding site (**H**) (initial and final poses in salmon and navy blue sticks, respectively). Transmembrane helices have different colors, and the lateral movement of the helices during the molecular dynamics simulations are reported in light blue

All four compounds displayed affinity for the MOR. Metonitazepyne and protonitazepyne affinity were approximately two and five times higher than that of fentanyl, but the two metabolites showed lower/marginal affinity; binding energy and K_i_ are reported in Table 2. RMSD over time during the last 20 ns of the MD simulations, i.e., when the steady state was reached, were 1.67 ± 0.19, 2.61 ± 0.07, 0.82 ± 0.05, and 1.21 ± 0.63 Å for metonitazepyne, protonitazepyne, 5-amino-metonitazepyne, and 5-amino-protonitazepyne (Supplemental Fig. S2), increased RMSD together with decrease in relative error suggesting high activation capacity. Initial and final binding poses of the four compounds within the binding site are displayed in Fig. 3.

The various amino acids of the receptor involved in binding and the interaction types with the four compounds are reported in Table 3.

**Table 3:** Amino acids of the MOR transmembrane helices (TM) involved in interactions with metonitazepyne, protonitazepyne, and main metabolites. The type of interaction is described with a symbol: □, H bond; ◊, dipole-induced dipole; ○, π stacking; ●, CH-π; ▪, CH-CH

Amino acid	Compound			
	Metonitazepyne	5-Amino-meto-nitazepyne	Protonitazepyne	5-Amino-proto-nitazapyne
His54	○		□	
Ser55	●	□	○	
Tyr75 (TM1)			●	
Val143 (TM3)	▪			
Ile144 (TM3)	▪			
**Asp147 (TM3)**	**◊**	**□**	**◊**	◊
Tyr148 (TM3)	●		●	○
Phe152 (TM3)	●			
Phe221 (TM5)				●
Leu232 (TM5)				●
Lys233 (TM5)	□		◊	
Trp293 (TM6)				
His297 (TM6)				□
**Val300 (TM6)**	**○**	●	**□**	●
Trp318 (TM7)			□	
Ile322 (TM7)			▪	
Gly325 (TM7)			◊	
Ile322 (TM7)				
Tyr326 (TM7)			□	

## Discussion

### Protonitazepyne and metonitazepyne metabolism

Good correlation was found between protonitazepyne-positive urine and hepatocyte incubations, with *N*-butanoic acid-protonitazene (B16) and *O*-despropyl-protonitazepyne glucuronide (B2) being predominant. Although all metabolites detected in protonitazepyne-positive blood were also identified in vitro, 5-amino-protonitazepyne (B3) was the main metabolite in blood but marginal in incubations. This discrepancy between urine and blood results was previously observed with other nitazenes (Krotulski et al. 2021a; Taoussi et al. 2024), and may be explained by the faster urinary elimination of *O*-desalkyl and *N*-butanoic acid metabolites compared to nitro-reduced derivatives, with the former being more polar. Protonitazepyne signal was less intense than that of the main metabolites in both blood and urine, suggesting substantial metabolization. Noteworthy, nitazenes were shown to be metabolized by highly polymorphic enzymes (Jadhav and Fasinu 2024), and the metabolic profile likely varies depending on the time of sample collection after consumption. Analysis of multiple positive samples taken at varying time points after uptake is recommended to confirm the present results.

The in vitro metabolism of metonitazepyne was consistent with that of protonitazepyne and other structural analogs under the same incubation conditions (Berardinelli et al. 2024; Taoussi et al. 2024). Similar results are, therefore, expected in vivo, with *N*-butanoic acid metonitazene (A13) and *O*-despropyl-metonitazepyne glucuronide (A3) being major metabolites in urine, and 5-amino-metonitazepyne being major in blood.

We, therefore, propose 5-aminoprotonitazepyne and 5-amino-metonitazepyne in blood, and *N*-butanoic acid derivatives in urine, as biomarkers for detecting protonitazepyne and metonitazepyne consumption, respectively. The parent compounds should be additional analytical targets. Although useful for confirming consumption in hydrolyzed urine, *O*-dealkyl metabolites are not specific, as they are common to protonitazepyne and metonitazepyne, but also etonitazepyne (Vandeputte et al. 2022).

### In vitro opioid receptor activation of protonitazepyne and metonitazepyne

It is important to consider that the GTP G_i_ binding assay, like other in vitro approaches, is a limited model that does not measure the ultimate functional outcomes and does not account for various parameters such as cell types, receptor expression levels, or physiological states, which may impact drug activity. However, it provides quick results for clinical and forensic toxicologists to support clinical diagnoses, overdose management, and legal investigations.

Metonitazepyne and protonitazepyne were shown to act as full MOR agonists, with potencies approximately twofold and sevenfold higher than fentanyl, respectively. These findings are consistent with studies by De Vrieze et al. and Kozell et al., which demonstrated that *N*-pyrrolidine-substituted nitazenes, such as protonitazepyne, metonitazepyne, etonitazepyne, and isotonitazepyne, exhibit greater MOR potency compared to their classic *N*,*N*-diethyl-substituted analogs (Kozell et al. 2024; De Vrieze et al. 2024). Using different analytical approaches (β-arrestin2 recruitment assay, bioluminescent cAMP reporter assay, and [^35^S]GTPγS functional assay), the authors obtained similar values for protonitazepyne (EC_50_ = 0.09–0.94 nmol L^−1^, E_max_ = 93.8–198%) and metonitazepyne (EC_50_ = 9.32–18.2 nmol L^−1^, E_max_ = 95.3–174%); apparent potencies and efficacies may vary depending on the assay principle (analytical technique, point of signaling cascade assessed). These results suggest that both compounds, particularly protonitazepyne, may induce potent analgesic and euphoric effects, but also carry a significant risk of fatal respiratory depression and dependence.

Metonitazepyne and protonitazepyne also exhibited low potencies and only partial agonism at DOR and KOR, suggesting that their pharmacological effects are primarily driven by MOR activation. These results are comparable with the in vitro experiments by Kozell et al. (Kozell et al. 2024). The MOR selectivity may contribute to their high toxicity and abuse liability, due to the lack of counterbalancing KOR-mediated aversive signaling.

### In silico MOR docking of protonitazepyne, metonitazepyne, and their main metabolites

Considering the marginal effects of metonitazepyne and protonitazepyne at DOR and KOR, as demonstrated by in vitro receptor activation studies, in silico docking was performed only at MOR. Both compounds showed high affinity at the receptor, with K_i_ approximately twofold and fivefold higher than fentanyl, respectively. These results are consistent with the in vitro results by Kozell et al., who measured K_i_ of 0.92, 0.29, and 1.25 nM for metonitazepyne, protonitazepyne, and fentanyl at MOR (Kozell et al. 2024), i.e., ratio of 2.2, 2.3, and 2.9 when compared to in silico K_i_. The in silico receptor docking results are also in line with the present in vitro receptor activation data, hinting a strong correlation. Although receptor affinity does not equal activation, and further investigation with multiple analogs is warranted, docking simulations may be used to estimate the activity of nitazenes and their metabolites, helping to guide experimental research.

Both compounds docked to the classic opioid binding pocket. At this site, ligand binding induces a conformational shift in the TM helices, with TM6 undergoing an outward movement, which creates a cavity for G-protein coupling and downstream signaling (Xie et al. 2022). In particular, ionic interactions between the protonated opioid form and Asp147 (TM3) are crucial for MOR docking and serve as a key determinant of binding affinity and activation. Morphine-type opioids typically bind Tyr148 (TM3) via H bonding and π–π stacking and Trp318 (TM7) via π–π stacking and hydrophobic interactions. In contrast, fentanyl-like opioids tend to interact more prominently with Val300 (TM6) via hydrophobic and van der Waals interactions and His297 (TM6) (analogous to rat His319) via H bonding. Tyr148, Val300, and His297 are important for stabilizing the opioid within the pocket, while Trp318 is critical for morphine-like ligands (Xu et al. 1999; Vo et al. 2021; Xie et al. 2022). In our experiments, although metonitazepyne and protonitazepyne exhibited different interaction patterns, with 9 and 11 amino acids involved, respectively, both compounds interacted with Asp147, Tyr148, and Val300, suggesting a similar binding mode to MOR compared to classic opioids; both also interacted with extra-helical residues His54 and Ser55. RMSD over time indicated good stability at the receptor, and the two compounds showed comparable oscillation values after 90 ns. A translational movement of TM1, 2, 3, and 7 was observed upon binding, with protonitazepyne inducing more significant movements than metonitazepyne, indicating stronger binding.

In vitro MOR activation and in silico docking results were congruent, and binding simulations were, therefore, used to predict metabolite activity. Considering the results of the metabolite identification experiments, which showed that the 5-amino derivatives of metonitazepyne and protonitazepyne are predominant in the bloodstream and therefore the most pharmacologically relevant metabolites, MOR docking was simulated for A1 and B3. Both compounds showed affinity for MOR, but their K_i_ values were substantially higher than those of their parent compounds and even morphine, especially for 5-amino-metonitazepyne, whose affinity for MOR appeared marginal. The patterns of oscillations during MD simulations confirmed this trend. Notably, both compounds interacted with Asp147 and Val300 (and also Tyr148 for 5-amino-protonitazepyne), but showed limited interactions with other amino acids classically involved in opioid binding. TM translational movements also were limited upon binding. Together, these results indicate that 5-amino-metonitazepyne and 5-amino-protonitazepyne may be pharmacologically active, albeit to a substantially lesser extent than their parent compounds. The results are consistent with the in vitro experiments by Vandeputte et al. showing that 5-amino-isotonitazene is active, but more than 200 less potent than its parent compound isotonitazene (Vandeputte et al. 2021).

## Conclusion

Protonitazepyne and metonitazepyne are MOR-selective full agonists, presenting significant health risks through CNS and respiratory depression. Both compounds undergo extensive metabolism, with 5-amino derivatives in blood and *N*-butanoic acid derivatives in urine, as major metabolite biomarkers of consumption. In vitro MOR activation and in silico docking results were congruent, and metabolite activity can, therefore, be anticipated through in silico receptor docking, avoiding the need for laboratory experiments, which are often costly, time-consuming, and dependent on the synthesis or commercial availability of analytical standards. Docking simulations showed that 5-amino-protonitazepyne and 5-amino-metonitazepyne might be active, although much less than their parent compounds. MD might become a critical tool to keep pace with the highly dynamic NPS market, and these preliminary results warrant further investigation.

## Supplementary Information

Below is the link to the electronic supplementary material.

Supplementary file1 (PDF 261 KB)

Supplementary file2 (PDF 336 KB)

Supplementary file3 (PDF 640 KB)

Supplementary file4 (PDF 247 KB)

## Data Availability

The original contributions presented in the study are included in the article and supplemental material. Further inquiries can be directed to the corresponding author.
