# Supplementary material for: Human metabolism and pharmacological profiling of protonitazepyne and metonitazepyne, two highly potent nitazenes: prediction of main metabolite activity based on µ-opioid receptor docking simulations
Source: Arch Toxicol. 2025 Oct 31;100(2):543–56. doi: 10.1007/s00204-025-04163-4 (PMC12886327; doi:10.1007/s00204-025-04163-4)

Supplementary Figure S1

Extracted-ion chromatograms of metonitazepyne and protonitazepyne, and metabolites identified in human hepatocyte incubates and protonitazepyne-positive postmortem blood and urine from a case of intoxication. Mass tolerance, 5 ppm

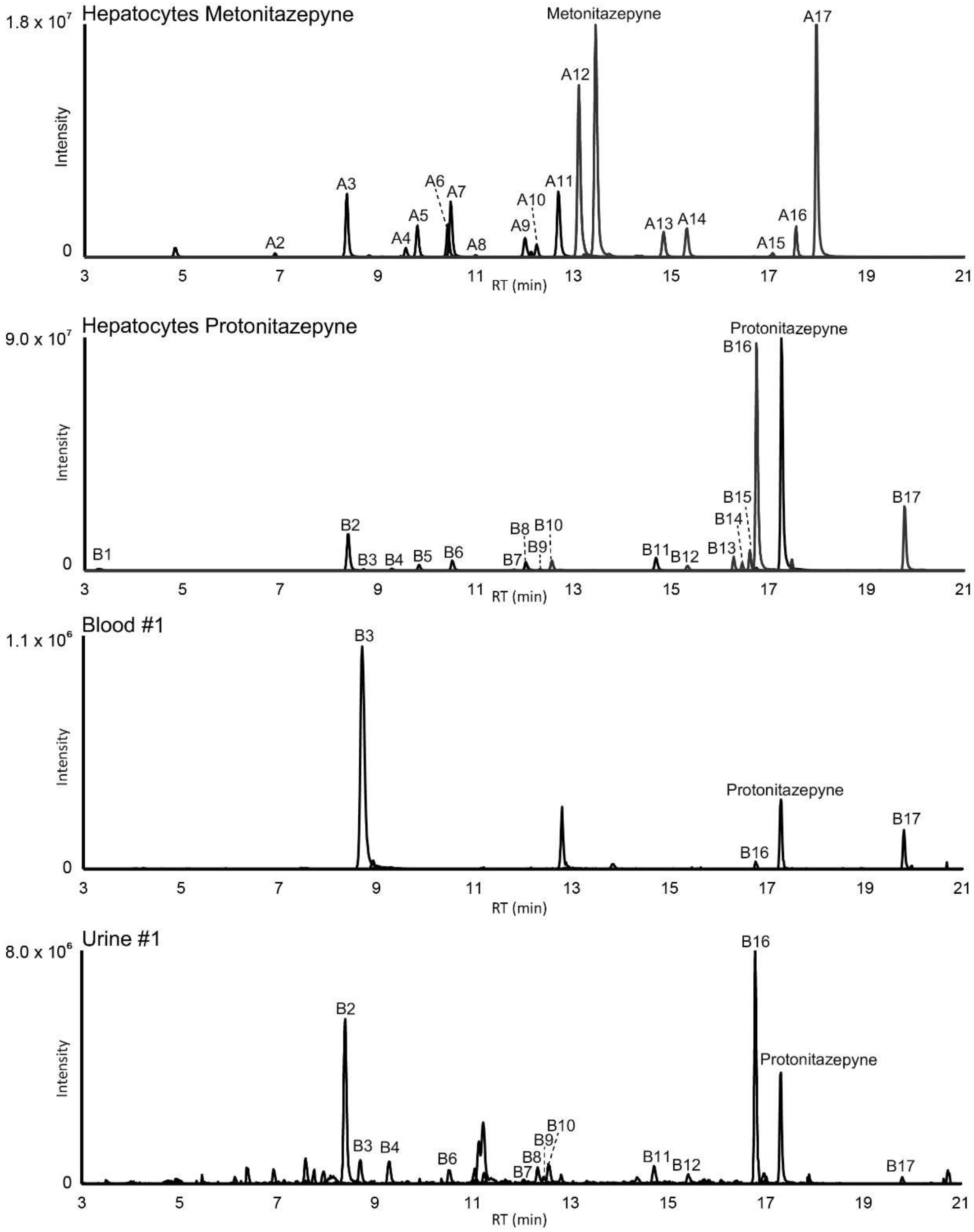

Supplement: Supplementary file 1 — Supplementary file1 (PDF 261 KB) [file 204_2025_4163_MOESM1_ESM.pdf]
