# Supplementary material for: Human metabolism and pharmacological profiling of protonitazepyne and metonitazepyne, two highly potent nitazenes: prediction of main metabolite activity based on µ-opioid receptor docking simulations
Source: Arch Toxicol. 2025 Oct 31;100(2):543–56. doi: 10.1007/s00204-025-04163-4 (PMC12886327; doi:10.1007/s00204-025-04163-4)

Supplementary Figure S2

Binding mode of BU72, a known μ-opioid receptor agonist: Comparison between crystallographic data (displayed in orange) and focused docking (blue); nitrogen and oxygen atoms are shown in blue and red, respectively (A). Representative model of ligand-μ-opioid receptor complex within the membrane; Proteins are shown in blue ribbons, lipids in cyan surface, water in red quick surface, and Na^+ and Cl^- ions in purple and green VdW spheres, respectively. The position of the ligand is highlighted in yellow VdW spheres (B). Root Means Square Deviation (RMSD) over simulation time of the four compounds (C). Mapping of the binding site, highlighted in surface; the most hydrophilic amino acids are displayed in navy blue, the most hydrophobic in green (D).

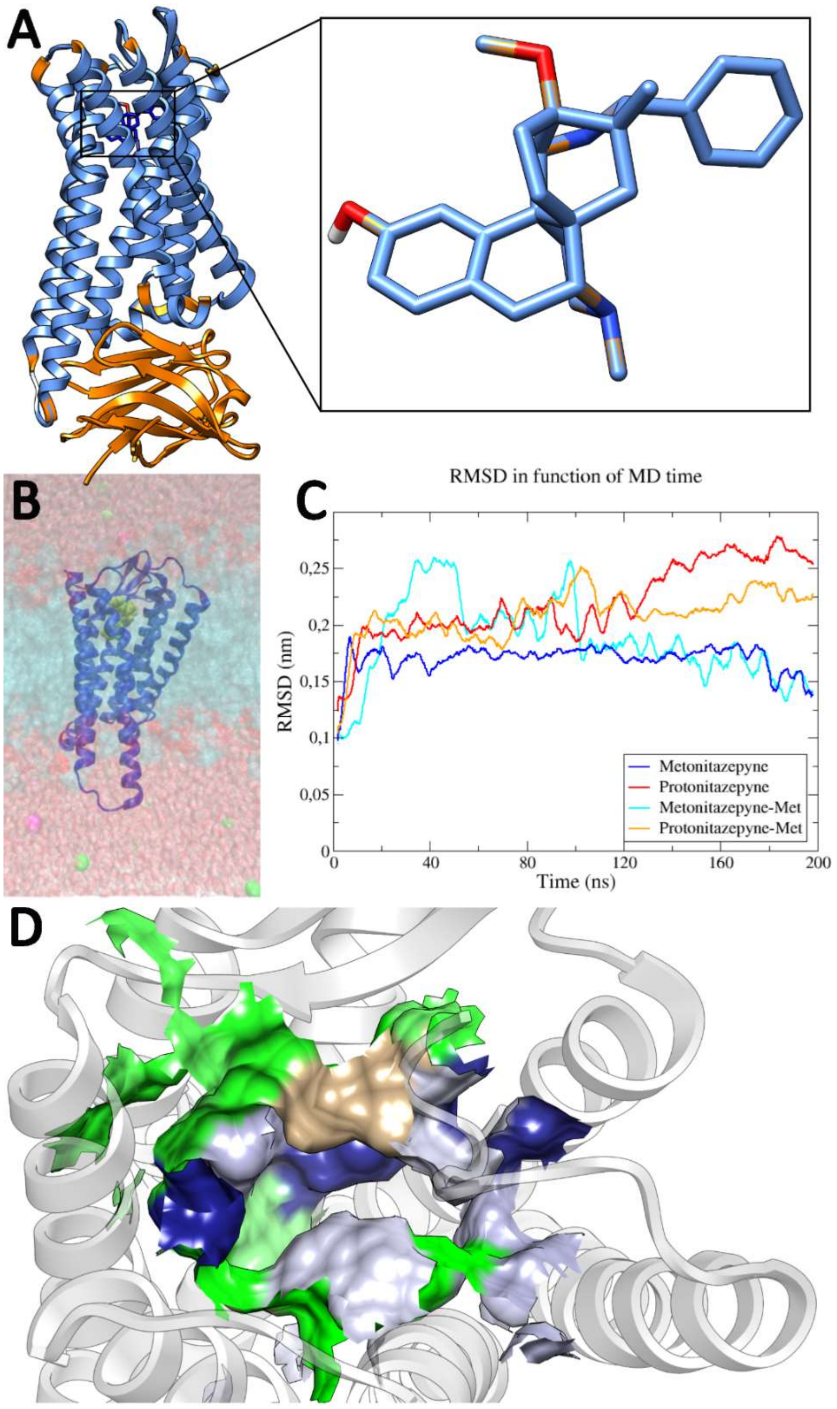

Supplement: Supplementary file 2 — Supplementary file2 (PDF 336 KB) [file 204_2025_4163_MOESM2_ESM.pdf]
