# Supplementary material for: Human metabolism and pharmacological profiling of protonitazepyne and metonitazepyne, two highly potent nitazenes: prediction of main metabolite activity based on µ-opioid receptor docking simulations
Source: Arch Toxicol. 2025 Oct 31;100(2):543–56. doi: 10.1007/s00204-025-04163-4 (PMC12886327; doi:10.1007/s00204-025-04163-4)
Supplement: Supplementary file 3 — Supplementary file3 (PDF 640 KB) [file 204_2025_4163_MOESM3_ESM.pdf]

Table S1-1. Inclusion list used during liquid chromatography-high-resolution tandem mass spectrometry (LC-HRMS/MS) for protonitazepyne metabolite identification.

**Protonitazepyne**			
**Transformation**	**Elemental composition**	**$[M+H]^+$ *m/z***	**$[M-H]^-$ *m/z***
**Parent**	**$C_{23}H_{28}N_4O_3$**	**409.2234**	**407.2089**
**-3C-6H**	$C_{20}H_{22}N_4O_3$	367.1765	365.1619
**+3C+2H+6O**	$C_{26}H_{30}N_4O_9$	543.2086	541.1940
**+O**	$C_{23}H_{28}N_4O_4$	425.2183	423.2038
**-2H+O**	$C_{23}H_{26}N_4O_4$	423.2027	421.1881
**+2H+O**	$C_{23}H_{30}N_4O_4$	427.2340	425.2194
**+2O**	$C_{23}H_{28}N_4O_5$	441.2132	439.1987
**+2H-2O**	$C_{23}H_{30}N_4O$	379.2492	377.2347
**-3C-6H+O**	$C_{20}H_{22}N_4O_4$	383.1714	381.1568
**-3C-8H+O**	$C_{20}H_{20}N_4O_4$	381.1557	379.1412
**+6C-8H+7O**	$C_{29}H_{36}N_4O_{10}$	601.2504	599.2359
**-3C-4H+O**	$C_{20}H_{24}N_4O_4$	385.1870	383.1725
**-3C-6H+2O**	$C_{20}H_{22}N_4O_5$	399.1663	397.1517
**-2H+2O**	$C_{23}H_{26}N_4O_5$	439.1976	437.1830
**-4C-7H-N+O**	$C_{19}H_{21}N_3O_4$	356.1605	354.1459
**-4C-6H**	$C_{19}H_{22}N_4O_3$	355.1765	353.1619
**+3C+2H+7O**	$C_{26}H_{30}N_4O_{10}$	559.2035	557.1889
**+2H+2O**	$C_{23}H_{30}N_4O_5$	443.2289	441.2143
**+3O**	$C_{23}H_{28}N_4O_6$	457.2082	455.1936
**+6C+10H+7O**	$C_{29}H_{38}N_4O_{10}$	603.2661	601.2515
**+6C+8H+8O**	$C_{29}H_{36}N_4O_{11}$	617.2453	615.2308
**-3C-4H-2O**	$C_{20}H_{24}N_4O$	337.2023	335.1877
**+2C+4H-O**	$C_{25}H_{32}N_4O_2$	421.2598	419.2453
**-7C-13H-N+O**	$C_{16}H_{15}N_3O_4$	314.1135	312.0990
**+2C+H-N+7O**	$C_{25}H_{29}N_3O_{10}$	532.1926	530.1780
**-4C-7H-N+2O**	$C_{19}H_{21}N_3O_5$	372.1554	370.1408
**-7C-12H**	$C_{16}H_{16}N_4O_3$	313.1295	311.1150
**-O**	$C_{23}H_{28}N_4O_2$	393.2285	391.2140
**-3C-6H-O**	$C_{20}H22N_4O_2$	351.1816	349.1670
**+2H-O**	$C_{23}H_{30}N_4O_2$	395.2442	393.2296
**-3C-4H-O**	$C_{20}H_{24}N_4O_2$	353.1972	351.1827
**-3C-6H+3O+S**	$C_{20}H_{22}N_4O_6S$	447.1333	445.1187
**+4O+S**	$C_{23}H_{28}N_4O_7S$	505.1751	503.1606
**-4C-7H-N+4O+S**	$C_{19}H_{21}N_3O_7S$	436.1173	434.1027

Table S1-2. Inclusion list used during liquid chromatography-high-resolution tandem mass spectrometry (LC-HRMS/MS) for metonitazepyne metabolite identification.

Metonitazepyne

Transformation	Elemental composition	$[M+H]^+$ *m/z*	$[M-H]^-$ *m/z*
**Parent**	**$C_{21}H_{24}N_4O_3$**	**381.1921**	**379.1776**
**-C-2H**	$C_{20}H_{22}N_4O_3$	367.1765	365.1619
**+5C+6H+6O**	$C_{26}H_{30}N_4O_9$	543.2086	541.1940
**+O**	$C_{21}H_{24}N_4O_4$	397.1870	395.1725
**-2H+O**	$C_{21}H_{22}N_4O_4$	395.1714	393.1568
**+2H+O**	$C_{21}H_{26}N_4O_4$	399.2027	397.1881
**+2O**	$C_{21}H_{24}N_4O_5$	413.1819	411.1674
**+2H-2O**	$C_{21}H_{26}N_4O$	351.2179	349.2034
**-C-2H+O**	$C_{20}H_{22}N_4O_4$	383.1714	381.1568
**-C-4H+O**	$C_{20}H_{20}N_4O_4$	381.1557	379.1412
**+6C-8H+7O**	$C_{27}H_{32}N_4O_{10}$	573.2191	571.2046
**-C+O**	$C_{20}H_{24}N_4O_4$	385.1870	383.1725
**-C-2H+2O**	$C_{20}H_{22}N_4O_5$	399.1663	397.1517
**-2H+2O**	$C_{21}H_{22}N_4O_5$	411.1663	409.1517
**-4C-7H-N+O**	$C_{17}H_{17}N_3O_4$	328.1292	326.1146
**-4C-6H**	$C_{17}H_{18}N_4O_3$	327.1452	325.1306
**+5C+6H+7O**	$C_{26}H_{30}N_4O_{10}$	559.2035	557.1889
**+2H+2O**	$C_{21}H_{26}N_4O_5$	415.1976	413.1830
**+3O**	$C_{21}H_{24}N_4O_6$	429.1769	427.1623
**+6C+10H+7O**	$C_{27}H_{34}N_4O_{10}$	575.2348	573.2202
**+6C+8H+8O**	$C_{27}H_{32}N_4O_{11}$	589.2140	587.1995
**-C-2O**	$C_{20}H_{24}N_4O$	337.2023	335.1877
**+2C+4H-O**	$C_{23}H_{28}N_4O_2$	393.2285	391.2140
**-5C-9H-N+O**	$C_{16}H_{15}N_3O_4$	314.1135	312.0990
**+2C+H-N+7O**	$C_{23}H_{25}N_3O_{10}$	504.1613	502.1467
**-4C-7H-N+2O**	$C_{17}H_{17}N_3O_5$	344.1241	342.1095
**-5C-8H**	$C_{16}H_{16}N_4O_3$	313.1295	311.1150
**-O**	$C_{21}H_{24}N_4O_2$	365.1972	363.1827
**-C-2H-O**	$C_{20}H_{22}N_4O_2$	351.1816	349.1670
**+2H-O**	$C_{21}H_{26}N_4O_2$	367.2129	365.1983
**-C-O**	$C_{20}H_{24}N_4O_2$	353.1972	351.1827
**-C-2H+3O+S**	$C_{20}H_{22}N_4O_6S$	447.1333	445.1187
**+4O+S**	$C_{21}H_{24}N_4O_7S$	477.1438	475.1293
**-4C-7H-N+4O+S**	$C_{17}H_{17}N_3O_7S$	408.0860	406.0714
