# Supplementary material for: Human metabolism and pharmacological profiling of protonitazepyne and metonitazepyne, two highly potent nitazenes: prediction of main metabolite activity based on µ-opioid receptor docking simulations
Source: Arch Toxicol. 2025 Oct 31;100(2):543–56. doi: 10.1007/s00204-025-04163-4 (PMC12886327; doi:10.1007/s00204-025-04163-4)
Supplement: Supplementary file 4 — Supplementary file4 (PDF 247 KB) [file 204_2025_4163_MOESM4_ESM.pdf]

Table S3. Compound Discoverer processing settings for generating proto- and metonitazepyne putative metabolites

**Phase I reactions**	Desaturation (–2H → ø)<br>Dihydrodiol formation (ø → +2H +2O)<br>Hydration (ø → +2H +O)<br>Ketone formation (–O → +2H)<br>Nitro Reduction (Intermediate 1) (O → ø)<br>Nitro Reduction (Intermediate 2) (O → H2)<br>Oxidation (ø → +O)<br>Oxidative deamination to alcohol (–2H –N → +H +O)<br>Oxidative deamination to ketone (–3H –N → +O)<br>Reduction (ø → 2H)<br>Metonitazepyne *O*-demethylation (–C –H3 → +H)<br>Nitazepyne *N*-dealkylation (–C4 –H8 → +H2)<br>Protonitazepyne *O*-depropylation (–C3 –H7 → +H)
**Phase II reactions**	Acetylation (–H → +2C +3H +O)<br>Cysteine conjugation (–H → +3C +6H +N +2O +S)<br>Cysteine-Glycine conjugation (–H → +5C +9H +2N +3O +S)<br>Glucuronide conjugation (–H → +6C +9H +6O)<br>GSH conjugation (–H → +10C +15H +3N +6O +S)<br>Methylation (–H → +C +3H)<br>Sulfation (–H → +H +3O +S)
**Max number of dealkylations**	3
**Max number of phase II reactions**	2
**Max number of all steps**	5
**Adducts**	$[M+H]^+$<br>$[M–H]^–$
